# Around the Hospitals

**Published:** 1893-09-23

**Authors:** 


### AROUND THE HOSPITALS.

Notice to the Managers of Institutions.—Special space is now reserved for the insertion of notices of hospital meetings and festivals. The Editor requests that all notices may reach The Hospital Office, 428, Strand, at least one week before the date of each meeting.

**Ingham Infirmary, South Shields.**—The annual report of this institution shows that its affairs, financially and otherwise, are pursuing a satisfactory course, and the Committee and management generally may be congratulated on the harmony which evidently reigns within its walls. Warm recognition was accorded by the Committee to the services of the medical staff throughout the year, and the Chairman bore testimony to the manner in which all the hospital officials worked together for the good of the institution. A proposal has been made to increase the number of the hon. medical staff, and the question is to be considered and a report made to a special meeting of Governors.

**Ancoats Hospital, Manchester.**—Again, from yet another hospital, comes the same story of failing subscriptions and an adverse balance. The good work done by the Ancoats Hospital deserves better recognition at the hands of the public. A pleasanter fact to be recorded is the addition of another endowed bed, the givers being three children—Frida Reiss and May and Conna Hargreaves—who, having made £110 from the proceeds of a bazaar, wish to devote the money to a cot in the children's ward, and hope in a few years' time to raise another fund in a similar manner to carry on its maintenance. The generous gift of £1,000 has been received from Mr. James Jardine, president of the institute, with the object of providing the patients with a permanent recreation-ground, and the same kind friend has requested the committee to make this plot of ground bright and attractive, furnish it with seats, and hand the account to him. During the past year important additions have been made to the hospital in the shape of new nurses' and servants' dining-rooms. Special appeals are made for funds to enable the committee to build a second convalescent home, the present one at Wilenslow being available for women only, and similar accommodation for men being urgently required. The number of patients treated increases every year, reaching a total during 1892-3 of 13,904. We heartily concur in the resolution adopted at the annual meeting: "That this hospital merits a much larger share of public support than it has hitherto received."

**Miller Hospital and Royal Kent Dispensary, Greenwich.**—The annual report of the general committee of this charity shows that an ever-increasing demand is being made upon its resources. Of in-patients, 263 were treated in the year just ended, as against 243 in 1892; while 3,267 accident cases were attended, compared with 2,435 last year, and a corresponding increase appears in other departments. Under these circumstances it is not surprising to find that applications for admission have frequently to be refused owing to lack of accommodation, and the committee state that a scheme is in preparation for the enlargement of the hospital, a scheme which it is to be hoped will meet with every support and encouragement. During the past year an eye department has been organised, and has proved helpful to some hundreds of patients. The committee "gratefully acknowledge the untiring services of the honorary chaplain, the Rev. Brooke Lambert," and their hearty thanks are also given to the medical and surgical staff, the secretary, matron, and staff generally "for their assiduity and attention to their duties." Amongst other gifts the committee acknowledge a thankoffering of 100 guineas from Mr. Job Ashton.

